# Predominant role of *Haemophilus influenzae* in the association of conjunctivitis, acute otitis media and acute bacterial paranasal sinusitis in children

**DOI:** 10.1038/s41598-020-79680-6

**Published:** 2021-01-08

**Authors:** Ya-Li Hu, Ping-Ing Lee, Po-Ren Hsueh, Chun-Yi Lu, Luan-Yin Chang, Li-Min Huang, Tu-Hsuan Chang, Jong-Min Chen

**Affiliations:** 1 Department of Pediatrics, Cathay General Hospital, Taipei, Taiwan; 2 Department of Pediatrics, National Taiwan University Hospital and National Taiwan University College of Medicine, Taipei, Taiwan; 3 Department of Laboratory Medicine, National Taiwan University Hospital and National Taiwan University College of Medicine, Taipei, Taiwan; 4 Department of Internal Medicine, National Taiwan University Hospital and National Taiwan University College of Medicine, Taipei, Taiwan; 5 Department of Pediatrics, Chi-Mei Medical Center, Tainan, Taiwan

**Keywords:** Bacterial infection, Bacterial pathogenesis, Infectious-disease epidemiology

## Abstract

*Haemophilus influenzae* is a predominant pathogen for conjunctivitis, acute otitis media and acute bacterial paranasal sinusitis in children. We undertook this study to investigate the possible association among these diseases. Children younger than 18-year-old with a diagnosis of bacterial conjunctivitis plus acute otitis media and/or acute bacterial paranasal sinusitis during 2009–2018 were included. Sampling for bacterial cultures was obtained from the lower palpebral conjunctiva and/or ear discharge with cotton-tipped swabs. A total of 67 children were recruited and the age was 29.5 (± 22.4) months in average. Fifty-seven children had conjunctivitis–otitis media syndrome and eight of them had a concurrent diagnosis of acute paranasal sinusitis. Ten children had conjunctivitis and acute paranasal sinusitis simultaneously. Clusters in household were observed in 50.7% children. Most common isolates were *Haemophilus influenzae* (70%), *Moraxella catarrhalis* (18%), and *Staphylococcus aureus* (8%). Antibiotic resistance rate of *H. influenzae* was 80% for ampicillin, 18% for amoxicillin–clavulanate, and 11% for the second or third-generation cephalosporins. Apart from well-known conjunctivitis–otitis media syndrome, acute paranasal sinusitis may also be linked to conjunctivitis with a similar pathogenic process. Simultaneous presence of these infections may guide the choice of empiric antibiotics toward *H. influenzae*.

## Introduction

Purulent conjunctivitis, acute otitis media, and acute bacterial paranasal sinusitis are common infectious disease during childhood. Common etiologies of acute otitis media and acute sinusitis include *Streptococcus pneumoniae*, *Haemophilus influenzae*, and *Moraxella catarrhalis*^1–5^. They are also predominant pathogens of bacterial conjunctivitis in children^6^.

The relationship between purulent conjunctivitis and acute otitis media was first noticed in 1965^1^. Another study conducted by Bodor found that 73% of the 132 children with purulent conjunctivitis had acute otitis media simultaneously^7^. Conjunctivitis–otitis media syndrome, also called conjunctivitis–otitis syndrome, was then denominated in 1982. The incidence of conjunctivitis–otitis media syndrome ranged from 32 to 73% in children with bacterial conjunctivitis^6,7^. Children younger than 2 years were the vulnerable population to this syndrome, and *H. influenzae* was the most correlative pathogen^1,7–9^.

After launching of pneumococcal and *H. influenzae* type b vaccines, studies suggest that nasopharyngeal colonization proportion of non-typeable *H. influenzae* (NTHi) is increasing^10^. The global burden of non-invasive infections, including otitis media, acute bacterial paranasal sinusitis, conjunctivitis, and pneumonia, owing to NTHi is also high currently^11^. Antimicrobial susceptibility of isolated bacteria has regularly been recorded in the Clinical Microbiology Laboratory of National Taiwan University Hospital (NTUH) comprehensively. Antimicrobial resistance rate of *H. influenzae* has increased dramatically in recent years. Ampicillin susceptible rate of *H. influenzae* decreased from 46% in 2011 (between January and June) to 39% in 2018 (between January and June). Amoxicillin–clavulanate susceptibility rate also declined from 97 to 91% within the same period (unpublished data). This is in accordance with our previous report^12^.

There were few investigations on possible changing features of conjunctivitis–otitis media syndrome in recent years, especially with respect to changing antibiotic susceptibility of *H. influenzae*. The relationship between conjunctivitis, acute otitis media, and acute bacterial paranasal sinusitis has not been elucidated, although *H. influenzae* is a common pathogen for all these three disorders. The present study was initiated to investigate the etiology, clinical features, antibiotic susceptibility patterns, and possible association of conjunctivitis, acute otitis media, and acute bacterial paranasal sinusitis in children.

## Results

### Biological data and clinical manifestations

A total of 67 children was recruited with ages ranging from 5 months to 10 years. The mean age was 29.5 (± 22.4) months with a median of 26 months (IQR 10–45). Forty-six (69%) patients were ≤ 3 years old. Demographic data and bacterial culture results are described in Table 1. The mean age of the children in which a particular pathogen was identified varied significantly (*p* = 0.01). Children with *H. influenzae* infection were the youngest, while the mean age of children with *S. aureus* infection was the oldest. The male-to-female ratio was 1.6. Fifty-seven children had conjunctivitis–otitis media syndrome. Six children had unilateral conjunctivitis and 60 children had bilateral conjunctivae involvement. On the other hand, 27 children had unilateral otitis media and 29 had bilateral infection. Affected eyes of one child and ear lesion site of one child were not recorded. Eight (14%) children with conjunctivitis–otitis media syndrome also had acute bacterial paranasal sinusitis at the same time. Ten (14.9%) children, including six boys and four girls, had a diagnosis of conjunctivitis accompanied with acute bacterial paranasal sinusitis. All of them had bilateral ocular lesions.

**Table 1:** Bacteriological culture results of conjunctiva discharge in children diagnosed of conjunctivitis with acute otitis media and/or acute bacterial paranasal sinusitis.

Gender and age	*H. influenzae* (N = 55)	*M. catarrhalis* (N = 14)	*S. pneumoniae* (N = 4)	*S. aureus* (N = 6)
%	70%	18%	5%	8%
Mean age ± SD*	26.6 ± 18.7	27.6 ± 18.6	43 ± 24.2	55.7 ± 40
**Age group, n**				
< 1y	15	4	1	0
1–2y	13	2	0	1
2–3y	11	2	0	2
3–4y	6	4	0	0
4–5y	6	1	2	0
> 5y	4	1	1	3
Male, n (%)	31 (56%)	12 (86%)	3 (75%)	4 (67%)

*In months.

Both conjunctival swab and ear discharge cultures were obtained for one child with ear drum perforation. Remaining children received conjunctival swab for bacterial culture only. Culture results of 55 (82%) patients showed single pathogen, while 12 (18%) samples showed multiple pathogens. Seven (10%) children had co-infection of *H. influenzae* and *M. catarrhalis*. The most common pathogen was *H. influenzae* (n = 55, 70%), followed by *M. catarrhalis* (n = 14, 18%), *S. aureus* (n = 6, 8%), and *S. pneumoniae* (n = 4, 5%). *H. influenzae* was also the leading pathogen contributing to acute otitis media (46/57, 81%) and acute bacterial paranasal sinusitis (15/18, 83%).

**Figure 1:**
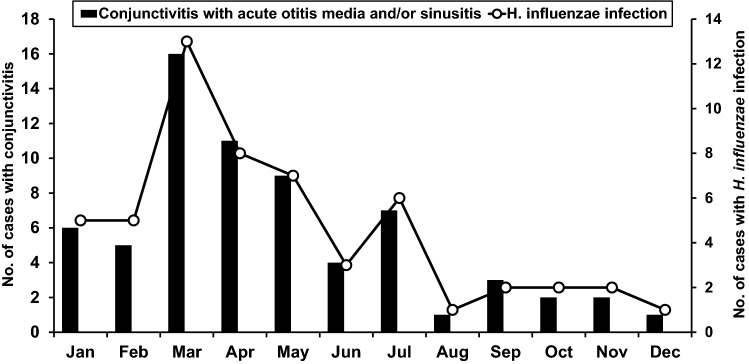
Occurrence of conjunctivitis with acute otitis media and/or acute bacterial paranasal sinusitis and *Haemophilus influenzae* infection by months. The black bar shows the number of cases with conjunctivitis and acute otitis media and/or acute bacterial paranasal sinusitis in each month. The solid line with circle shows number of cases with conjunctiva culture results of *H. influenzae*.

Conjunctivitis accompanied with acute otitis media and/or acute bacterial paranasal sinusitis is most prevalent in March and April, corresponding to spring season in Taiwan (Fig. 1). Clusters in household were observed in 34 (51%) patients. At least one family member of these children had conjunctivitis and respiratory tract infection with or without fever within one week of illness of index patient. Three families had two children with a diagnosis of conjunctivitis plus acute otitis media and/or acute bacterial paranasal sinusitis contemporary.

Seven (10%) children had underlying diseases including biliary atresia, congenital heart disease, Down syndrome, bronchial asthma, prematurity, and cleft palate. Only four (6%) patients needed hospitalization. A 2-year-old boy was admitted for one week due to a complication of preorbital cellulitis. The other three children were hospitalized for contemporary bronchopneumonia or dehydration due to poor oral intake. In addition, a 10-year-old boy had ophthalmological complications of keratitis and corneal ulcer during follow-up. Oral cefixime was administrated initially and the conjunctiva discharge culture yielded methicillin-susceptible *S. aureus*.

One child had recurrent conjunctivitis–otitis media syndrome one and a half years later. Another child had recurrent acute otitis media with complication of complex febrile convulsion required hospitalization two weeks after the initial episode.

### Antibiotic susceptibility

Amoxicillin–clavulanate resistance rate of *H. influenza*e increased gradually and the average resistance rate in this study was 18% (ten of 55 isolates) (Table 2). Excluding one missing data, all nine isolates were β-lactamase-positive-amoxicillin-clavulanate resistant strains. *H. influenzae* isolated form three children had multiple antimicrobial resistance including ampicillin, amoxicillin–clavulanate, cefixime, cefpodoxime and cefuroxime. Most of the infected children received antibiotic treatment with amoxicillin–clavulanate. Other commonly used antibiotics were the second- or third-generation cephalosporins. *H. influenzae* strains isolated from girls tended to have a higher resistant rate against amoxicillin–clavulanate (29%, seven of the 24 isolates) than boys (10%, three of the 31 isolates), although the difference is not statistically significant (*p* = 0.06). Resistant rate of *H. influenzae* did not correlate with contact or cluster history (four of 27 [14.8%] versus six of 28 [21.4%]; *p* = 0.53), nor with underlying diseases (nine of 50 [18%] versus one of five [20%]; *p* = 0.91).

**Table 2:** Antimicrobial susceptibilities of *Haemophilus influenzae* isolates recovered from conjunctiva or ear discharge using the disk diffusion method^*^.

Agent	No. of isolates tested	No. (%) of indicated susceptibility	
		Susceptible	Resistant
Ampicillin	54	11 (20)	43 (80)
Amoxicillin-clavulanate	55	45 (82)	10 (18)
Chloramphenicol	54	50 (93)	4 (7)
Cefixime	54	52 (96)	2 (4)
Cefpodoxime	50	48 (96)	2 (4)
Cefotaxime	53	53 (100)	0 (0)
Cefuroxime	54	48 (89)	6 (11)
SXT	54	17 (31)	37 (69)

SXT, Trimethoprim/sulfamethoxazole.

*One child received both conjunctiva and ear discharge culture examination, and both yielded *Haemophilus influenzae* with the same antimicrobial susceptibility pattern.

## Discussion

*H. influenzae* is the most frequently isolated pathogen in pediatric bacterial conjunctivitis, responsible for 44–68% of all cases. *S. pneumoniae* and *M. catarrhalis* cause 7–44% and 1–6% of cases, respectively^6^. The link between bacteria conjunctivitis and otitis media has been well established since the report by Bodor in 1982 that emphasized the important role of *H. infleunzae*^7^. To our knowledge, our study is the first report demonstrating the link among bacterial conjunctivitis, acute otitis media and acute bacterial paranasal sinusitis. Eight (14%) children with conjunctivitis–otitis media syndrome also had acute bacterial paranasal sinusitis. Ten (14.9%) children had purulent conjunctivitis and acute bacterial paranasal sinusitis without acute otitis media. Nine (90%) of the conjunctival discharge culture yielded *H. influenzae*. In summary, a quarter of the participants had conjunctivitis accompanied with sinusitis with or without acute otitis media in our study. Besides, the participants recovered from acute conjunctivitis when receiving antibiotic treatment for acute otitis media and/or paranasal sinusitis. These findings suggest that there may be a “conjunctivitis–otitis media–sinusitis syndrome” that shares a same pathogenic process as conjunctivitis–otitis media syndrome.

Howie and Schwartz et al. had proven high correlations of nasopharyngeal cultures with cultures of middle ear effusion^13,14^. It is plausible that a single microbiological entity may feature in these conditions due to the continuity of the mucosa in the upper respiratory tract and indeed the co-occurrence of conjunctivitis–otitis media syndrome^15^. Because H. influenzae may enter conjunctival sac, middle ear, and paranasal sinus at the same time, our data shows that there is quite a tight association between conjunctivitis and otitis media, and between conjunctivitis and sinusitis. However, the chance of involvement of all the three infection sites concurrently is low. This is the reason why the number of children with all the three infections are low in present study. Although the pathogenesis remains to be elucidated, we postulated that rhinitis caused by viral infection or allergic inflammation of the upper respiratory tract may lead to edema of the ostia of Eustachian tubes, nasolacrimal ducts and paranasal sinuses, resulting in obstruction and bacterial superinfection. Eventually, conjunctivitis, otitis media and sinusitis may develop simultaneously^4,7^. One study on ferret did show that otitis media and sinusitis may occur after challenge of influenza virus followed by *S. pneumoniae*^16^. Because *H. influenzae* is a common inhabitant of nasopharynx^4,17^, it is reasonable for it to become a predominant pathogen for all the three infections. Such an association has been partly mentioned once in one previous report from Taiwan showing that *H. influenzae* prevailed in children with concurrent acute otitis media and acute bacterial paranasal sinusitis^18^.

One thing unusual is that *S. pneumoniae* is the most prevalent pathogen for isolated acute otitis media and isolated acute bacterial paranasal sinusitis, followed by *H. influenzae*^11,19^. Our study and previous studies all demonstrate that *H. influenzae* exceeds *S. pneumoniae* to become the most prevalent pathogen when otitis media and/or sinusitis are associated with conjunctivitis^7–9^. Universal 13-valent pneumococcal conjugate vaccination was started in 2013 in Taiwan^20^. Although the use of pneumococcal conjugate vaccine may decrease the proportion of infections caused by *S. pneumoniae*, a predominant role of *H. influenzae* has been noted before the pneumococcal conjugate vaccine era. A recent study in Taiwan showed that *S. pneumoniae* remains the most common etiology of acute otitis media^18^.

In contrast to otitis media and sinusitis, *H. influenzae* is the most common etiology of bacterial conjunctivitis^7,11^. On the other hand, most *S. pneumoniae* strains causing bacterial conjunctivitis lack capsules and are not typeable^21,22^. These facts imply that when compared with *S. pneumoniae*, *H. influenzae* is in general more virulent to conjunctiva, and is less virulent to middle ear and paranasal sinuses. Although unencapsulated *S. pneumoniae* may be more virulent to conjunctiva than encapsulated serotypes, unencapsulated strains have a low ability to cause otitis media and sinusitis. This is the reason why *S. pneumoniae* is not prevalent in “conjunctivitis–otitis media–sinusitis syndrome.”.

Some studies show that neuraminidase produced by *H. influenzae* and unencapsulated *S. pneumoniae* may disrupt heavily sialylated mucosal surface and degrade surface mucin, facilitating the attachment of bacteria and establishment of an infection^22,23^. However, the exact virulent factor of “conjunctivitis–otitis media–sinusitis syndrome” remained to be investigated.

Many *H. influenzae* strains may produce biofilm which leads to failure of antibiotic treatment and liability of recurrence^24,25^. One French study showed that biofilm production is low among *H. influenzae* strains associated with conjunctivitis–otitis media syndrome^26^. The recurrence rate of acute otitis media is 4% (2/57) in our study, which is relatively low comparing with other studies^27,28^. Small sample size might be one of the explanations.

Crowded environment including many siblings at home or daycare center might facilitate the transmission of *H. influenzae*^29^. Clusters of purulent conjunctivitis and/or otitis media were observed in 47% of families in one study in 1982^7^. We have a similar finding of household clusters in 34 (51%) patients. Clustering of conjunctivitis within families also highlights the possibility of *H. influenzae* as the most possible offending pathogen of associated otitis media and sinusitis.

Prevalence of β-lactamase-producing *H. influenzae* differs widely worldwide. Studies have reported percentages of β-lactamase-positive *H. influenzae* between 10 and 25% in most regions, including South Africa, Europe, USA, Canada, Central America, and South America)^11^. In some regions (Taiwan, Vietnam, Japan, South Korea), β-lactamase-positive strains account for up to 55% of *H. influenzae* with a high prevalence of β-lactamase-negative ampicillin-resistant strains and β-lactamase-positive-amoxicillin-clavulanate resistant strains^11,30,31^.

The present study shows an alarming high ampicillin resistance of 80%, and a high amoxicillin-clavulanate resistant rate of 18% for *H. influenzae*. Except for one missing strain, all amoxicillin–clavulanate resistant isolates were β-lactamase-positive-amoxicillin-clavulanate resistant trains. Combination of β-lactamase production and presence of penicillin-binding protein 3 mutations is thought to be the mechanism of resistance^31,32^.

A high ampicillin resistance rate (around 55–60%) of *H. influenzae* has been observed for a long time in Taiwan since 2001^12,33^. Amoxicillin–clavulanate resistance rate of *H. influenzae* is low in western countries, and has not been well described. This is in contrast to reports from Taiwan, Japan, and South Korea that showed an amoxicillin–clavulanate resistance rate of 10–25% among *H. influenzae* isolates^12,18,30,31^.

Our study has several limitations. First, medical records of 13 participants did not mention domestic cluster history. Whether similar disease occurred among other kindergarten attendees or not was only described in three children. Therefore, the transmission rate due to clusters in household or school might be underestimated. Second, previous antibiotic exposure at other medical institutions could not be ascertained. We can not clarify its possible association with the high antibiotic resistance rate observed in present study. Finally, our study was performed in a single tertiary hospital. The small sample size may lead to a variation toward the true incidence of responsible pathogens and antimicrobial susceptibility rate. Nevertheless, our study provides updated information about the association of *H. influenzae*, conjunctivitis, acute otitis media and bacterial paranasal sinusitis. This is an important information for all physicians taking care of children all over the world.

Apart from well-known conjunctivitis–otitis media syndrome, acute bacterial paranasal sinusitis is also relevant to conjunctivitis with a similar pathogenic process. The appearance of “conjunctivitis-otitis media-sinusitis syndrome” gives hints to offending pathogens and such an information is important for the choice of empiric antibiotics. The most common etiology for the syndrome is *H. influenzae.* Ampicillin, and amoxicillin–clavulanate in some areas, are not very active against this pathogen. There is an imminent need of developing effective vaccines to protect against non-typeable *H. influenzae* infections.

## Methods

### Study design

Children younger than 18 years old with a diagnosis of bacterial conjunctivitis plus acute otitis media and/or acute bacterial paranasal sinusitis during January 2009 and December 2018 were included. Acute otitis media is defined as bulging, erythema, opacity and reduced mobility of the tympanic membranes^7^. Occurrence of acute otitis media and conjunctival hyperemia with purulent discharge simultaneously corresponds to conjunctivitis–otitis media syndrome. A diagnosis of acute bacterial paranasal sinusitis was made when a child with an acute respiratory tract infection presents with one the following: (1) worsening course, i.e., worsening or new onset of nasal discharge or fever after initial improvement; (2) severe onset, i.e., concurrent fever (temperature ≥ 39 °C) and purulent nasal discharge for at least three consecutive days^34^.

Sampling for bacterial cultures was taken from the lower palpebral conjunctiva with cotton-tipped swabs for all participants. We also obtained middle ear effusion for bacterial culture if patients had otorrhea. The specimens were plated onto trypticase soy agar on 5% sheep blood agar plates (BAP) and chocolate agar (Becton–Dickinson Microbiology Systems, Sparks, MD, USA) that were incubated at 35 °C in an aerobic atmosphere (BAP) or 5% CO_2_ (chocolate agar plates) for 24–48 h. Bacteriae were identified via standard method and confirmed by VITEK 2 (BioMérieux, Marcy l'Etoile, France) or BRUKER BIOTYPER matrix-assisted laser desorption/ionization time-of-flight mass spectrometry system (Bruker Daltonik GmbH, Bremen, Germany). *H. influenzae*, *S. pneumoniae*, *Staphylococcus aureus*, *M. catarrhalis*, and *Streptococcus pyogenes* were considered as correlative pathogens responsible for the infections.

We reviewed the patients’ biological data such as sex, age and past medical history, clinical manifestations, bacterial culture results of conjunctiva and/or ear discharge, and treatment regimens. All research followed relevant regulations and Helsinki guidelines. The institutional review board of National Taiwan University Hospital approved this study. Waiver of the informed consent was also approved by the institutional review board of National Taiwan University Hospital because this retrospective study involved no more than minimal risk to participants and would not adversely affects the rights of participants.

### Antimicrobial susceptibility testing

Antimicrobial susceptibility testing for *H. influenzae* was conducted using the disk diffusion test that was performed in the Clinical Microbiology Laboratory in NTUH. Isolates were interpreted as susceptible, intermediate, or resistant to tested agents, including ampicillin, amoxicillin-clavulanate, cefuroxime, and cefixime, etc., according to the interpreted criteria recommended by the Clinical and Laboratory Standards Institute (CLSI) guidelines^35^. β-lactamase production was also tested.

### Statistical analysis

ANOVA, Student’s t test or Mann–Whitney U test was used to examine differences among continuous variables. Chi-square test was used for categorical variables. All statistical analysis via SPSS version 22 is two-tailed and *p* < 0.05 is considered statistically significant.

## Data Availability

The data that support the findings of this study are from the National Taiwan University Hospital and are not publicly available.
